# Supplementary material for: Seeing through the forest: The gaze path to purchase
Source: PLoS One. 2020 Oct 9;15(10):e0240179. doi: 10.1371/journal.pone.0240179 (PMC7546910; doi:10.1371/journal.pone.0240179)
Supplement: Attachment — Submitted filename: Response to Reviewers-PONE-D-20-14107R1.docx [file pone.0240179.s001.docx]

**Response to reviewer comments**

**Reviewer #1:** This is my review of the manuscript, entitled “Seeing through the forest: The gaze path to purchase.” The authors present an eye-tracking study on 92 participants examining, in a 3 (display size) x 2 (price) within-subjects design, the link between the gaze sequence in which individuals viewed a display and their hypothetical choice of an unbranded plant product. While I am not able to evaluate the authors’ machine learning approach, my expertise lies in eye-tracking research, when applied to business settings. In that sense, I enjoyed reading the present manuscript, and I think the authors have a well-crafted initial submission. Therefore, based on my feedback provided below, my reviewer recommendation is to invite the authors for a minor revision. I start by delineating the larger topical concerns and areas for improvements, after which I continue with some minor suggestions to improve the readability and contribution of this piece.

| MAJOR POINTS: | Response |
| --- | --- |
| In connection to the authors’ theorizing and discussion on gaze aversion, I was missing some notes on whether and how this construct is related to, or clearly distinct from peripheral vision, which plays an important role in consumer choice situations. For example, how does the gaze aversion conceptualization differ from the account proposed by Wästlund et al. (2018; references provided in the end of this review)? This is important given that the authors write at several places that consumers often ignore some visual cues. | Our study should be considered clearly distinct from any investigations of peripheral vision. We are interested in the deliberate look-away fixations, not peripheral vision (which does not have a fixation). While peripheral vision is important to many other aspects of life, in product choice we are more interested in the deliberate effort to collect useful information for choice through the narrow foveal angle: a fixation. Furthermore, it is the disengagement with stimuli input that constitutes the LATD. Visually, humans can process stimuli *more accurately* through a fixation and cognate on that information whereas peripheral vision may not provide as much or as precise information. It is the disconnect from the input of that information that constitutes the look-away. The Wästlund et al. (2018) study showed that peripheral vision helped a subject find a product category, not a specific product, and helped eliminate prospective choices. Our work focuses on how the product choice is made from deliberately focused-upon set of seen alternatives (and deliberate disconnect from input periodically). In light of this reviewer’s comment, we included a new paragraph (beginning at line 248) within the gaze aversion section to further contextualize and differentiate the LATD concept from peripheral vision and the work of Wästlung et al. (2018). Also, we used this comment to expand of future lines of inquiry (line 538). |
| While eye-tracking studies in lab settings have their merits, they lack a certain degree of ecological validity. This should be acknowledged in the limitations section of the authors’ manuscript | Thank you for bringing our attention to this oversight. We have added a paragraph on limitations to the study at line 527. |
| I found the authors’ exposition to be a bit front-heavy, particularly the review of literature section, which I think can be written more succinctly. | This reviewer had more eye-tracking expertise compared to the second reviewer. For broad-reaching journals such as PLOS 1, selecting literature for inclusion or exclusion can be challenging. The second reviewer presented an opposing perspective to this one, commenting that the “study needs to be much better contextualized within the visual cognition literature rather than only using references to consumer psychology.” Thus, we elected to add some citations to meet that request. |
| When it comes to the authors’ contribution and significance statements, I think they should make more of an effort in order to not over-sell their findings. For example, they note that “few of the studies investigated the gaze paths in which visual cues were accessed and no studies investigated whether that relationship predicted purchase” (p. 4, last paragraph); that their “goal is to better understand the sequence of cues that help get products off the shelf and into the cart” (p. 5, first paragraph); and imply that they look at actual product choices and purchase decisions. Strictly speaking, however, all these claims are inaccurate, as the authors are solely examining hypothetical choice in a rather artificial lab setting (see my ecological validity point above). Please be precise. | Thank you for bringing the need for this clarification to light. We investigated product choice in the lab which may be indicative of purchase intention or choice in a retail setting. In the manuscript, we reworded “purchase” to “choice”. The lab setting is an ideal starting point for investigation before attempting to replicate findings in a less controlled environment. If we found a relationship between gaze sequence and/or LATD in the lab for choice, perhaps these relationships exists in the field for purchase. |
| I think the authors should report a 3 (display) x 2 (price) within-subjects ANOVA on the 11-point likelihood of buying scale, both as a validity check and as a way to potentially boost their managerial implications. Certainly, it would be interesting for a retail owner to know if there is a main effect of display size, such that, for instance, people indicate greater buying likelihood in a display with a larger (vs. smaller) number of product options. Similarly, it would be interesting, as a validity check, to see if participants’ buying likelihood were greater in the presence (vs. absence) of price discounts (with corresponding effect sizes), and to explore whether display size interacted with price to influence participants’ buying likelihood. Please implement these analyses or provide very solid arguments as for why such details are not included (arguments of the type “fell beyond the purpose of our study” is not enough in my view, but I will not hijack the story). | Thank you for this comment. We added a two-way repeated measures ANOVA to evaluate simultaneously the effect of two within-subject factors (display size and price) on likelihood of buying scale. Please find new Table 1 in the “Sample and Protocol” section.  We also added **Figure A** in the “Sample and Protocol” section to show mean likelihood-to-buy scores (LTB) for different display sizes and price settings. It indicates two main factors, display size and price, both affect participants’ LTB. Additionally, it shows the evidence that the display size interacts with price to influence participants’ degree of LTB.  The reviewer’s comments are also helpful in the construction of future work, building on this study. We did not intend to test the response to the sale price, but had a regular and sale price to replicate what might be seen in a real retail plant display. |
| The authors provide arguments supporting their findings on product expertise and previous annual purchases of plants. However, virtually no arguments at all appear for the finding that race (white) helped improve the model’s predictive accuracy, which left me with the question “why?” Please elaborate. | Purchase and plant-related activities in the U.S. historically has been an activity participated in largely by more affluent white consumers. This is evidenced in nearly three decades of research conducted by the National Gardening Association (see <https://gardenresearch.com/>) also documented in Dennis, J.H. and B.K. Behe. 2007. Evaluating the Role of Ethnicity on Gardening Purchases and Satisfaction. HortScience. 42(2):262-266. However, more recent NGA studies show the gap between white and persons from other ethnic backgrounds closing as participation among non-white consumers increases. We added this text to the manuscript (beginning at line 486). |
| MINOR POINTS: |  |
| Please specify the design (within-subjects) in the abstract | Corrected on line 26. |
| Please correct the claimed non-student sample in the abstract, as the main text in the manuscript seems to suggest that some students were also included in the study. | While some students may have been included in the sample, subjects were recruited from a pool containing both students and non-students. We corrected this on lines 26 and 299. |
| For the sales figures presented on p. 2 (final paragraph), the authors may want to have a more recent reference than 2016. Relatedly, they may want to add the fact that retail sales from physical stores still account for 90-95% of total retail sales (e.g., Otterbring & Lu, 2018). | We thank the reviewer for indicating this more recent citation and included it on line 41 and to the citations. |
| At p. 4 (first paragraph), I got confused, as the authors start by stating that the number of visual cues at the POP (point-of-purchase) can be OVERWHELMING at times, after which they state that their main goal is to study the relationship between gaze sequences and product choice for MINIMALLY packaged, UNBRANDED products. | We apologize for the confusion. The overwhelming choice set may cause cognitive overload which could lead to a need for disengagement from stimuli input. Still, the vast majority of studies involving consumer choice (both hypothetical and real) utilize packaged goods. The novelty of both minimally packaged and unbranded goods adds a different dimension to the literature. Furthermore, since these minimally packaged goods are slightly different, it may create an environment in which consumers may want to evaluate multiple products (to choose the best one?) as opposed to packaged goods which may have multiple facings, but show identical information with invariable quality (for the same branded item – SKU). In the case of identical branded products, a consumer can assess quality relatively quickly whereas for the minimally packaged good, scrutiny may need to be made to assess quality and the sheer number of cues can be overwhelming. We added this information to the study limitations (lines 534-535). In a future study we could include packaged goods (e.g. fertilizer, etc.) to see whether there are differences in gaze patterns. |
| When reviewing visual attention and purchase likelihood, the authors may want to add some recent eye-tracking references using hypothetical choice as the focal dependent variable (e.g., Ballco et al., 2019; Otterbring et al., 2020; Otterbring & Shams, 2019; Tórtora et al., 2019). | We appreciate the suggestions and incorporated those citations into the beginning of the literature review. |
| At p. 6 (first paragraph), the authors indicate some inconsistencies in findings, but they do not specify how these inconsistencies are manifested. Please clarify. | We revised the section on top down and bottom up processes to address this concern. The inconsistencies were seen between macro-level studies (e.g. wayfinding) and micro-level studies (e.g. product selection) which begins on line 115. |
| The real, imagined, or implied presence of others tend to influence individuals’ visual attention (e.g., Lie & Yu, 2017; Milani et al., 2019; Wong & Stephen, 2019). Therefore, I think further methodological details are needed in the manuscript with respect to whether an experimenter was present in the same room as the participant(s) during the study and how many individuals that attended the eye-tracking sessions simultaneously. | Thank you for this helpful comment. We added “Each subject proceeded from one display to the next unaccompanied using a progressive start to the first display with no other participants viewing displays. Two researchers were in the same room, but out of the visual line of sight while one subject participated in the study” to lines 277-280. |
| Did all the signs displayed in the study use the same color scheme on and around the letters? | Yes, we added this information to line 270. The signs used black Calibri font either size 88 (non-price information) or 40 (price information) on white paper. |
| Please clearly indicate the sampling frequency and accuracy of the eye-tracking device used. | The sampling frequency was 50 Hz the 20 ms sampling intervals. This was added to the methods section on lines 301-302. The binocular accuracy was 0.62^o^. |
| At p. 16 (last paragraph), the authors state that CEN was identical for one model and lower for four of the five remaining models after adding the consumer attributes, after which they state that overall accuracy was identical with and without the addition of consumer data for two of the six models. This seems contradictory – was it one or two models that were not improved after addition of the consumer attributes? Please clarify. | We changed the evaluation metrics to F1-score as suggested by the second reviewer. We used the F1-micro score and F1-macro score. For the multi-class task, the F1-micro score is equivalent to overall model accuracy. Please find the updated Table 1 in results section (now labeled Table 4).  Table 5, in the results section, was added to summarize the best predictive model in the former Table 1 (now labeled Table 4), under two F1-scores, respectively. It indicates that for less complex displays (with lower price and smaller display sizes, considering consumer attributes improves the predictive accuracy; But for more complex displays (with larger display sizes, adding look away information when constructing 3mer (3mer-with-LATD in model 3 and model 4) have better predictive performance. |
| Please present the zero-order correlations between the included consumer attributes: expertise, being (vs. not being) white, and prior plant purchase. | The zero-order correlations between three consumer attributes can be found in Table 3 in the results section. |

In sum, I think the authors have an interesting study and my evaluation as a reviewer is that they may have the chance of getting their work published, in case they meticulously address all points raised above. I wish them good luck in their future work on this topic and I hope that my comments were helpful.

**Reviewer #2:** This study investigates the relation between overt attention and purchase choices (i.e., buying a plant) in a real-world scenario using 3D Tobii glasses. Participants’ gaze positions are monitored while asked to choose one of plant out of different many, and each is tested in six different conditions, which result from a manipulation of set size (6, 12, 24 plants), and the price band (low, high). From the eye-movement responses, they seem to consider the frequency of the first three fixations, and together with survey data of their consumer preferences, train a set of different random forest classifiers aimed at predicting which plant will be chosen. The classifiers seem to display a good accuracy for the small set-size (72%) and their performance improve when trained with survey data and a feature of the eye-movement defined as Look Away to Decide (LATD), which represents fixations outside of any Area of Interest.

It is interesting to read about the application of eye-tracking technology in different disciplines, and I truly appreciate the effort of building computational models based on eye-movement responses to make predictions in real-life contexts. The authors have also provided the data and scripts to run the analyses which is a nice and honourable aim. However, overall, the study falls short on its methodology, theoretical underpinnings and implications for it to make a substantial contribution.

In what follows, I highlight my major concerns and a few minor other comments that the authors may want to take on board to improve their study.

| Major comments: | Response |
| --- | --- |
| (a) There seems to be confusion about what the theoretical contributions of this work are and especially what their novelty should be. Since the seminal work of Yarbus, 1967, it is widely known that eye-movement responses can successfully predict the task participants perform. The notion of task can be broadly construed to encompass several things among which also the target object that people may be attentively selecting. It is not surprising then, that when considering the first three fixations only (more later about why this may be a methodologically problematic choice) it may be enough to predict what object is receiving most attention, and so correlate with the final choice. So, I find this result, per-se, not novel nor striking. What’s also odd is that the authors claim that looking away from the object is a good predictor of choice, that’s counterintuitive as usually fixation time correlates with the amount of attention that a target got. Furthermore, even in the context of consumer psychology, there are other studies, some of which mentioned by the authors, showing already systematic relations between gaze behaviour and choice. | We appreciate the reviewer’s comments and seek to provide evidence to support that a sequence of gazes can be predictive of a specific task (product choice). While most eye-tracking studies are task-specific, few include product choice or purchase intention (see Huddleston et al., 2018 [54]. The theoretical contribution of the manuscript is that gaze *sequence* is predictive of product choice. We used groups or sets of three fixations (called k-mers or 3-mers) not the first three fixations. While looking away may at first blush appear to be counterintuitive, we strived to show that part of the decision process involves stimulus disengagement, potentially for time to cognate. None of the studies referenced in the present paper include disengagement metrics.  To facilitate understanding of all stimuli, areas of interest, and gaze paths, we created an Appendix A which is posted with other study materials in the Open Science Foundation repository at this anonymous link: https://osf.io/hykwu/?view_only=5e36084775af40218c6a1617b3337e3b  The appendix A includes an example of a display, the areas of interest, one subject’s fixations (showing some look-aways) to show the look-aways are deliberate not spurious. |
| (b) The other novelty seems to be that the inclusion of LATD and survey data improves the prediction accuracy of the models. However, the predictions of the models presented in Table 1 seem to be erratic across display types, and do not follow any clear theoretical logic as to why that’s the case. In fact, the best accuracy seems to be Model 3 for a display of 6 plants at low price (called J, 78%), which includes consumer attributes. At the same time, we also see that a display with 24 plants and a high price (Q) reaches an accuracy of 73% with Model 2, which is the one with LATD features but not consumer attributes. If both consumer attributes and LATD are included as training features (Model 4), we see an incredible drop in performance for the display Q (36%), while for the display P, also 24 plants and low-price, there is a much better accuracy (75%). The lack of consistency across displays, or between training data and achieved model performance casts some important doubts about the reliability of these classification results, so undermining any theoretical statements related to it. | The new Table 2 (now Table 6) should help clarify this. We show that there is a high predictive ability of the models when including consumer attributes and LATD, especially for the higher price (more complex) decision. The lower sale price should not require as much cognitive effort. There is no one size fits all model. People look at high and low price differently; complexity adds another layer of cognitive effort. |
| (c) LATD feature is tentatively argued to be reflecting gaze aversion. But, instead, it could simply relate to data quality and nothing else. In fact, it does reflect fixations falling outside all possible area of interests. I would be really surprised that participants actively engaged in a choice task would spend time looking at no informative areas of the display. Thus, it would be important to quantify how many fixations fall outside the area of interests, whether the percentage of such fixations varies (or it is stable) across participants and displays, etc. In practice, shed better light to the nature of such out of range fixations. In general, there is very little detail about eye-tracking data quality, which is much needed. Also, the argument of gaze aversion does not really fly, in my humble opinion, as the literature this hypothesis refers to involves human-human interaction, while here, it is a very different setting with no-animate agents taking parts beyond the participants. | Similar to a comment by Reviewer 1 who encouraged us to relate the look-aways to peripheral vision, we deliberately looked at the aversion of gaze. These are not errors in the data and additional information may be gathered from Appendix A posted in the Open Science Foundation repository link: https://osf.io/hykwu/?view_only=5e36084775af40218c6a1617b3337e3b  The reviewer may observe from the figures in Appendix A that conservative tight lines were drawn around the plants. Allowing for some reasonable margin of error, most of the fixations outside the AOIs appear to be deliberate and not equipment error. Table 6 (formerly Table 2) includes mean percentage of looking away fixation across participants for each display. It shows that looking-away fixations are quite stable for all displays, ranging from 31% to 40%.  We also computed the mean percentage of looking away fixation across all participants and all displays, ending up with mean value of 35% and standard deviation of 8%. It also indicates the looking-away fixation is quite stable across all participants and displays.  We based our hypotheses on the need humans sometimes have to disengage from continuous information input. We speculated that continuously looking at choices may not be helpful to the decision process, which is what our data support. |
| (c) The study needs to be much better contextualised within the visual cognition literature rather than only using references to consumer psychology. There are few claims or arguments about bottom-up vs. top-down processes, for example, that seem misleading, inaccurate or poorly referenced. I have added a few works on the topic at the end of this review, so that they may give the authors a starting lead to relevant literature. Line 106:108 “Attention drawn to a sudden loud noise, flashing lights, or an attractive person is the result of a bottom-up process, while finding a friend’s face in a crowd is the result of a top-down process.” There should be a mention of what drives bottom-up processes, e.g., low-level visual saliency, and clarify what is meant by top-down. Is it the role of the task, e.g., visual search as opposed to free-viewing? Why is looking for faces top-down, because of the stimuli (i.e., faces) or because of the task? Also, it is not clear how the current study taps into such a dichotomy. In fact, as far as I can see, the experimental design does not systematically manipulate any of the two types of mechanisms. There is a manipulation of set size, referred by the authors as displays, and price, but none of the two is systematically related to any of the results presented. As said in my previous context, the results from the modelling do not show any systematicity with respects to these two independent variables. | The first reviewer presented an opposing perspective writing, “found the authors’ exposition to be a bit front-heavy, particularly the review of literature section, which I think can be written more succinctly.” However, we elected to not reduce the literature cited and rather chose to address your concerns. We re-worded lines 103-128 and added some citations to better contextualize the study, as shown below: Attention to visual cues implies an awareness of the stimuli in the conscious mind, which is driven by both top-down and bottom-up processes [14,31]. In the visual cognition literature, bottom-up processes refer to stimulus driven attention [32] or low-level stimulus features that capture attention. Research on bottom up processes finds that visual attention is driven by salient stimuli features (e.g. contrast, color) and is mostly involuntary and reactive [33]. Top down processes refer to the conscious experience of visual stimuli [34], are context dependent [35] with visual attention being allocated based on the nature of the task [33,35]. That is, attention is drawn to relevant areas based on the task at hand (e.g. selecting a blue flower). The visual cognition literature suggests that in the absence of a task, bottom-up processes influence visual attention, but once the task tells us what to look for, it changes the way we look at stimuli [36]. In other words, in the absence of a task, visual attention might be driven by the most salient feature of the stimuli, i.e. driven by a bottom-up process, but once a task is assigned visual attention will be directed toward those features that fulfill the task.  Wedel and Pieters [14] apply the findings of visual cognition literature to visual attention to marketing. They posit that (marketing) stimuli salience and informativeness act in combination to affect attention. Aligned with the visual cognition literature, Wedel and Pieters [14] propose that bottom-up processes operate when consumers attend to salient features such as color or contrast and this attention is mainly involuntary. Top-down processes, as identified by Wedel and Pieters [14] refer to perceptions of marketing stimuli that could be influenced by consumer demographics, brand familiarity, expertise, involvement and attitudes or goals. Unlike tasks assigned in the visual cognition literature which have a correct/incorrect outcome [33,35] marketing study tasks are usually open ended (which brand would you buy) with no right or wrong answer. Outcomes are determined by consumer factors. For this study, we are interested in analyzing which, if any, consumer characteristics influence gaze pattern for plant displays. |
| (d) There is also a long-standing literature about eye-movement scan-patterns, starting from the seminal paper by Noton & Stark 1971, and about modelling, or measuring similarity, between them. The authors seem not to be fully aware about this bulk of literature, and I think instead that such literature should be better discussed. Also, their analysis of scan-patterns sounds a bit unusual. For what I have understood, from all possible fixations that each participant made during a trial, only the first three were kept, and they used a rather unconventional naming for it (i.e., 3-mers). There is a weak attempt to explain why that was done but it did not fully convince me. I think the authors should show what happened when the scan-patterns were fully considered, or at least show, what happens to the modelling results as they increase the number of data-points from 3 to N. More importantly, from their analysis I gathered that the sequential aspect of scan-patterns was not considered. It seems that the random forest classifiers were in fact trained using relative frequencies of fixating at the different AOI, if that’s the case, then I do not see how this data can be defined as a scan-pattern. Modelling a scan-pattern implies preserving its sequential aspect, and utilising methods that can maintain it untouched, e.g., Hidden Markov Models. They should perhaps refocus their analyses in terms of extracting eye-movement features, and that they decided to consider the first three fixations as those one carrying important information about the task. I also wonder whether they may look for other characteristics of the oculo-motor behaviour, it may well be that there are other types of features, object-based, best fit to their classification task. | The Noton & Stark (1971) signaled the beginning of investigating visual patterns. That study showed common patterns common among stimuli whereas our investigation seeks to find common in a viewed stimulus, not the identification of a sequence of gazes. Most of the literature is focused on tasks which have little relevance to product choice (i.e. pick the orange circle, etc.). Non-attendance literature is also expanding, but our focus was the disengagement or look away to facilitate predicting product choice.  Text has been added interspersed between lines 402-412 in the "Data Handling and Analysis" section to describe the motivation for the use of the k-mer data format. The new text better describes k-mers and the utility of the k-mer data format for gaze fixation seqeunces. The reviewer was unintentionally led to believe that 3-mers meant that we only made use of the first three fixations from each participant. A 3-mer is a three letter subsequence from a gaze fixation sequence. For the sequence " CACZYBYZZBYB", there will be ten 3-mers: CAC, ACZ, CZY, ZYB, YBY, BYZ, YZZ, ZZB, ZBY and BYB. We have added similar example in the text and have provided a longer example in as a supplemental table. The addition of this example (as well as an example in Appendix A) should also clear up several other concerns of the reviewer, but we have made other modifications to avoid confusion for other readers of this manuscript. For example, we now point out in the text how the use of 3-mers retains sequential events in the gaze sequence. Additionally, as stated in the "Data Handling and Analysis" section, when we used 4-mers for the analysis, the frequency of individual 4-mers would have been too sparse to allow the machine learning algorithms to make accurate predictions.  As the use of all possible 3-mers from each gaze fixation sequence did retain information regarding the entire gaze sequence, we feel that our use of the RF and SVM classifiers did make use of all information in the gaze sequences, and we do not feel that the use of an HMM is necessary. We had sufficient accuracy with RF and SVM classifiers that we have been able to gain novel insights into consumer behavior during the examination of a complex product display, and we are satisfied with these two classifiers.  We did not use HMMs because we were not looking to train a machine learning algorithm that would be used to predict the buying choices of future consumers in some other scenario. We were looking to use the machine learning algorithm to understand features (eye tracking paths [the 3-mers] and consumer history from the questionnaire) that would allow us to better understand the decision-making process of the study participants. As noted, HMMs are complex probabilistic models that do not lend themselves to understanding the features that are important in a successfully trained HMM. Therefore, an HMM was not used to predict purchasing decisions  We did not test other aspects of oculo-motor behavior as this is beyond our expertise. |
| (e) The justification of using Random Forest instead of any other methods seems a bit weak. The authors say, Line: 289-291: “We selected the RF machine learning approach because of its many properties for classifying a high-dimensional data set such as the present one, which would be impractical in the context of traditional linear models due to parameter estimation problems.” I am not sure why, for example, Support-Vector machine would not work for their purpose. SVM can be trained on a technically infinite high-dimensional feature space to predict a certain outcome variable. As the features their random forest classifiers were trained on are relative frequencies of looks to AOI and survey data, I guess that SVM would also here. I think, they should better motivate their choice, and show that their method of classification, i.e., random forest, would really give them a better F-score than some other method. By cross validating their predictions using different methods may help them also realise what may be generating variability across displays. | Thanks for your suggestion. We applied two machine learning algorithms, SVM and Random Forest. We also changed our evaluation metrics to F1-score as you suggested. Two F1 scores are included, F1-micro and F1-macro score. For this multiclass classification task, F1-micro score is equivalent to overall accuracy. Please find the updated Table 1 (now Table 5) in the results section.  New Table 5 also shows the best predictive model, under two F1-score, respectively. It indicates that for less complex displays (with lower price and fewer plants), considering consumer attributes improves the predictive accuracy; But for more complex displays (with more plants), adding looking away information when constructing 3mer (3mer-with-LATD in model 3 and model 4) have better predictive performance. |
| (f) Survey data is used to train the random forests, but it is never explained at length what is the data consisting of, how is it treated, and inputted. For example, I take that each participant contributed to a single data point per questionnaire, but there are many more eye-movement data points per display. It is crucial that the treatment of the data is much better elucidated, otherwise the reader cannot really follow and appreciate what was done in the modelling. | Each subject had visual metrics for each of 6 displays. In addition, their survey responses which included measures for plant expertise and demographics were merged with the data. Thus, we extracted the entire gaze sequence for each subject for each display and merged that with the responses to the survey questions. Demographic characteristics included age, household income in the year prior to the study, number of adults and children in the household, ethnicity, and gender. |
| (g) The task itself is severely underspecified. I am still not sure, even after reading the paper a few times, whether participants had always to make a choice to buy something or not. If not, it is not clear how many times they did not make any choice, and how were these trials treated. In general, it is never clear how many trials were used to train the classifiers. Even if the data is provided, there is the need for greater details about the treatment of the data, and better descriptive statistics. | We reworded the task in lines 303-308. The task was, “Point to the plant you would buy and verbally state (0-10) how likely you are to buy that plant?”. Each subject could not actually purchase a plant; we kept the plants in place throughout the study. |
| Minor comments: |  |
| It may be useful to give an example of the Area of Interests. There seems to be a lot of overlap between the plants in the display, so I wonder how boundaries are defined. Also, the plants further away in the display may receive less fixations just because of their relative position. | We created Appendix A to show the display format, outlines of areas of interest, and sample gaze sequence. True that plants further away from the viewer may receive fewer fixations, but this was not a focus of our study. |
| Improve the quality of the only Figure, which is rather low. Also, it is not clear what this Figure is depicting. I would suggest to add more Figures to aid the understanding of the readers. | We agree and added Appendix A to further demonstrate how the plants were arranged, how the areas of interest were drawn, and how gaze sequences were developed. |
| Line 283:284 “The next step was to investigate whether gaze 3-mers would be predictive of product choice. For that purpose, we computed, for each participant, the relative frequency in which each 3-mer occurred in the first 100 gaze events in their gaze sequence.” Not sure I understand this passage. I got that 3-mers are the first 3 fixations in a sequence, how can they be 100 gaze events? How did they derive this number? Are 3-mers, three area of interest? If so, how were these regions chosen if the display contains many more (i.e., a minimum of 7 and a maximum of 25 including out of range fixations)? | We considered the whole sequence, not the first 100 gazes. Thank you for indicating this error; we corrected it (please see lines 327-328).  The sequence, 3-mers, and areas of interest are further outlined in Appendix A available at https://osf.io/hykwu/?view_only=5e36084775af40218c6a1617b3337e3b. |
| Line 256:257 The authors mention a chocolate bars practice task, but it is never clear why was this done, and what did it entail. | It was not a plant and it was something that we imaged everyone would be familiar with and not cause undue stress, but was helpful to introduce the subjects to the task of product choice. We used chocolate bars to be an easy product choice task and laid them out horizontally on a table identical to the one on which the plants were placed. |
| Line 258:259. The participants have to verbally state how much from 0 to 10 would they like to buy a product. But, was not it a buy vs. no-buy binomial outcome variable that’s modelled? If it is a continuous variable, what are the random forest predicting? A preference score? If so, how is prediction accuracy then measured? | We collected the variable “choice” using the 11-point Juster Scale (continuous) and then converted to the binary variable choice/no choice. Each subject was asked to point to the plant they would be most likely to purchase, state how likely they would be to purchase the plant, and no choice was recorded as 0. All subjects were aware they could opt to buy no plant at each display. |
| Line 231:232 “Plants occupied approximately 0.12 m2, 0.25 m2, and 0.45 m2 of display space for the 6, 12, and 24-plant designs, respectively” The authors should provide measurements in terms of degree of visual angle, as the size of objects is relative to the distance of the observer. | We agree with the reviewer’s comment that the degree of visual angle can be important, especially when having subjects stand still in front of a stimulus. In the present study, subjects wore a portable eye-tracker and were permitted to be as close or far from the plants as they desired. Thus, calculating the visual angle would vary widely by subject, their height, and at which moment in the study we chose to calculate that angle. The visual angle can be aggregated better for static eye tracking studies conducted on computer screens. We attempted to simulate a real plant retail display but visually isolate it (as well as the subject) from spurious visual stimuli that might have drawn the gaze away from the task at hand. |
| Line 202: There seems to be a typo. | The last word of the sentence “decision” was on the next page. With the new line numbering, the word is showing on line 177. |
| Line 309:312 “Our final models focused on predicting consumer choice for plants selected by at least 10 consumers (two plants in six-plant displays and three plants in the 12- and 24-plant displays) and for the no-plant choice.” Obscure passage. This sounds like data dredging. It is unclear how many final data points contributed to the modelling. | If we did not have enough participants who selected the same plant (n<10), the model would be over-fitted. We consciously elected to model only choices where we had a sufficient n to investigate the effect of the 3-mers and consumer features on choice. In real life, some products would be selected more often than others; it was this phenomenon we sought to model.  We also add sample sizes of each display in line 393. |
| Line 323:324 “To determine which attributes had a strong impact on the plant choice, variable selection was performed using a series of F-tests.” The authors now mention feature selection, but it was never clear how many features were originally selected, and what were F-tests based on. | We added some clarification into the data analysis section. The original features were based on different models from model 1 to model 4. Then used ANOVA F tests to filter relevance of numerical features and reduce the high dimension feature space. |
| Table 1: There is what seems an arbitrary list of letters to identify the displays. Not sure what a reader could make of it. | The original list identified the display without using the potentially biasing first letters of the alphabet. We removed the labels from the tables and simply used a numerical sequence, plant number, and price. |
| Line 364: There is a mention of baseline, but it is not clear what the baseline is, and how it is computed. | We revised the wording on line 437 to show that the model is more predictive than chance (1/24) which we called a baseline. We also revised the model description in the data analysis section [Line 404-410]. |
| Line 400:403 There is a mention to EEG research but it feels quite tangential and off-topic. | Physiological metrics beyond gaze are used to predict choice; specifically we reference the Sundararajan et al. and Ravaja et al. studies which used EEG measures. Our goal is to show the comparison for predictive accuracy for our model was better than the former. We revised the wording to show a stronger relationship in lines 497-501. |
